# Effect of match load on perceived wellness in highly trained female football players

**DOI:** 10.1371/journal.pone.0321505

**Published:** 2025-04-21

**Authors:** Ivan Baptista, Andreas Alexandersen, Andreas K. Winther, Dag Johansen, Svein Arne Pettersen

**Affiliations:** 1 Department of Computer Science, Faculty of Science and Technology, UiT The Arctic University of Norway, Tromsø, Norway; 2 Centre for Research, Education, Innovation, and Intervention in Sport (CIFI2D), Faculty of Sport of the University of Porto, Porto, Portugal; 3 Department of Psychology, Faculty of Health Sciences, UiT The Arctic University of Norway, Tromsø, Norway; 4 School of Sport Sciences, Faculty of Health Sciences, UiT The Arctic University of Norway, Tromsø, Norway; Universidade de Aveiro Escola Superior de Saude de Aveiro, PORTUGAL

## Abstract

**Background:**

Exposure to match loads significantly affects physiological and psychological indicators and, consequently, players’ wellness. However, this information is still scarce in women’s football. Therefore, the aims of this study were twofold: a) to compare the wellness variation from matchday (MD) to two days post-match (MD+2); b) and to analyse the correlations between the players’ external load on MD and the self-reported wellness on the day after the match (MD+1) and MD+2.

**Methods:**

This retrospective cohort study included data from 22 weeks and 33 official matches from 18 professional and semi-professional female football players competing in the Norwegian top-tier. Signals for total distance, high-speed running distance (>16 km/h^-1^), sprint distance (>20 km/h^-1^), acceleration distance, and number of sprints were collected using a Global Positioning System. Sleep duration and four wellness subsets were included in this study: sleep quality, delayed onset muscular soreness, fatigue and stress levels. Individual models were run using the respective wellness variable as the dependent variable, with matchday as a predictor. Data was modelled using cumulative link regression models. The model allowed random slopes for subjects to account for repeated measurements. Correlation analysis was computed using Spearman’s rank correlations.

**Results:**

Our results from the cumulative link regression model suggest that fatigue increased on MD+1 (Estimate: 1.30; SE=0.16; p<0.001) and remained elevated on MD+2 (Estimate: 0.75; SE=0.15; p<0.001), when compared to MD. Sleep quality decreased on MD+1 (Estimate: -0.72; SE=0.14; p<0.001). Sleep duration decreased on MD+1 (Estimate: -0.70; SE=0.13; p<0.001) and on MD+2 (Estimate: -0.61; SE=0.13; p<0.001). Moderate correlations were observed on MD+2 between sleep duration and acceleration distance (0.32, p<0.001) and high-speed running distance (0.30, p<0.001).

**Conclusions:**

Competitive matches are associated with a disruption in the stability of the players’ sleep patterns and wellness. The results also suggest that univariate external load measures may not be strong enough to predict the players’ wellness status variation in the days following matches.

## Introduction

Women’s football is developing rapidly, and the number of women and girls playing organised football all over the world is rising to over 16.6 million, with a total of 3.9 million being registered players [1]. The increasing number of registered female players and competitions allowed women to train and compete under more professional circumstances [2]. Despite the rising interest in women’s football, studies investigating elite women football players account for only 15% of all football research published [3]. Most studies including women footballers have focused on injury prevention, physical performance, psychosocial factors, motivation and leadership [4], while information regarding fatigue development in matches and players’ wellness is still scarce.

Football has previously been defined as a psychophysiological phenomenon since the exposure to training and match loads (the product of the session’s volume and intensity) greatly affects physiological and psychological indicators and, consequently, players’ wellness [5]. Training and match load can be divided into external (physical work performed) and internal (physiological and psychological response) load and analysed through objective (e.g., Global Positioning System [GPS]) or subjective (e.g., questionnaires) measures. Monitoring training and competition load has become an essential part of understanding athletes’ individual responses to pursue the most efficient training and recovery strategies. These strategies may vary daily since previous research has demonstrated how external training load varies across a range of typical cycle lengths in professional women’s football [6]. Player monitoring encompasses not only the quantification of match and training demands but also the assessment of players’ readiness. Readiness, as defined by Beato et al. [7], refers to “the state of being fully prepared to train and perform physically and mentally”. Thus, according to the authors, the concept of wellness should be included in the readiness construct. This means that since health is a criterion of readiness, players’ state of preparedness to play depends on specific factors such as stress, sleep, delayed onset muscle soreness (DOMS), and fatigue.

An impairment of physical performance characterises post-football match fatigue during the days following the match and has several potential causes [8]. Previous research on female [9] and male football players [10] from different levels has reported that, after a competitive match, at least 72 hours are required to normalise muscle damage and inflammation, replenish muscle glycogen stores, and to achieve full neuromuscular recovery. This way, the combination of an appropriate amount of load and rest is paramount for better performance and to reduce the injury risk potential [11].

For a better weekly planning and daily management, the post-match recovery status of footballers has been investigated by resorting to self-reported measures of wellness [12]. When tracking post-match recovery, a sensitive tool for post-match fatigue is required [13]. Indeed, acceptable levels of consistency and sensitivity were previously reported for certain subjective measures, with correlations observed between these measures and the external training load from the day before [14]. Moreover, despite the existence of several athlete self-report measures (POMS, DALDA, REST-Q, TQR), many of these are often extensive and time-consuming, with faster, simpler and nonexhaustive tests being the most recommendable tools to quantify fatigue status in elite team-sport athletes [15].

In this regard, negative small-to-moderate correlations between wellness measures and locomotor activity demands were identified in a recent systematic review [16]. This evidence was further strengthened, and correlations were also reported between the wellness index and internal training load [17]. In particular, players’ wellness following match-play should be carefully monitored as this event often elicits the greatest stimulus of the microcycle [13]. Oliveira et al. [18], in a study with professional male football players, observed that wellness variables, including fatigue, sleep quality, stress and DOMS, remained similar across the entire week, except on the day after the match. In fact, according to previous research, ratings of fatigue and DOMS following a match have increased up to 42% and 39%, respectively [13]. Similarly, the highest rates of DOMS are reported 24–48 hours after exercise [19].

Practitioners acknowledge the relevance of sleep quality and quantity to overall health, recovery, and performance [20]. Despite the recognised importance of sleep as a fundamental component for physical and mental recovery, enhancing cognitive function and overall athletic performance [21], contradictory results have been reported regarding the association between training/match loads and sleep indices. While some studies report no impact of high-intensity sessions on sleep quality and sleep duration [22], others report associations between high external and internal loads with improved sleep indices [23].

Recently, in a study with female football players, Scott et al. [24] analysed the effect of external match load on players’ perceived wellness. However, only self-reporting ratings of fatigue and DOMS were examined, with no reference being made to the indicators of sleep and stress. Therefore, in order to provide further scientific information regarding the influence of match demands on players’ perceived wellness on the two days following the match, the specific aims of this study were twofold: a) to describe and compare the wellness variation from matchday (MD) to two days post-match (MD+2); b) and to analyse the correlations between the players’ external load on MD and the self-reported wellness on the day after the match (MD+1) and MD+2. Based on previous findings with female athletes [25], the following hypotheses were established: i) wellness variables and sleep duration vary significantly from MD to MD+1 and MD+2; and ii) high-speed metrics accumulated during MD correlate with the self-reported wellness and sleep variables analysed.

## Methods

The data to support the findings of this study can be found publicly available on the open science framework (https://osf.io/wcqkx/).

### Participants and experimental design

Following approval from the Norwegian Centre for Research Data (296155), written informed consent was obtained from 18 professional and semi-professional female players (23.3 ± 5.2 years of age) representing two teams in the Norwegian top tier (ten players from one team and eight from another) and classified as highly trained [26]. From March 2020, a prospective observational study was conducted collecting perceived wellness and external match load data from over 22 weeks and 33 official matches, totaling 175 match observations. The typical microcycle in these 22 weeks was characterized by six days between matches, with four to five training sessions and one to two days-off. Both MD+1 and MD+2 varied between days-off and training days throughout the data collection period. Players’ data were included if a player: a) played for the same team during the entire study; b) reported the wellness questionnaire at least once, and c) played >70 minutes of an official match. The final sample comprised a minimum of 3 and a maximum of 13 matches per player and an average of 6.88 matches per player. The median value was 5, and the interquartile range was 6.

Prior to the commencement of the study, ethical approval was sought from the Regional Committee for Medical and Health Research Ethics-Northern Norway (reference number 53884). However, since the data collection did not involve a biobank, medical, or health data related to illness, nor did it disrupt the normal operation of the players, the need for consent was waived.

### External load, wellness variables and sleep duration

External match load was collected using GPS STATSports Apex (Newry, Northern Ireland), with a sampling frequency of 10Hz. The validity and levels of accuracy (bias<5%) of this tracking system have been previously presented [27]. During matches, each player wore a tight vest with the GPS unit on the back of their upper body between the scapula as described by the manufacturer. The microsensor devices were activated 15 min prior to the start of each match in accordance with the manufacturer’s recommendations [28]

Raw data curation and pre-processing of speed and acceleration signals followed the same methodology of previous research [28]. Velocity thresholds applied for high-speed running distance (HSRD) (>16 km∙h^-1^) and sprint distance (SpD) (>20 km∙h^-1^) are in accordance with previous research on women’s football [29]. Acceleration distance (Acc_dist_) was defined as the distance covered with a positive or negative change in speed of more than ± 2.26m/s^-2^ finishing when the rate of Acc_dist_ reached 0 m/s^-2^ [30].

Players were asked to report their perceived wellness and sleep duration on MD, MD+1 and MD+2 on a linear 5-point Likert scale. The data quality, internal consistency and discriminative validity of this scale were assessed in previous research [31], where the authors suggested the use of the five-point scale version in future applications. A qualitative indicator, as proposed by McLean et al. [32], was used to assist players with reporting. Four wellness subsets were included in this study: sleep quality, DOMS, fatigue and stress levels. Sleep duration was also reported and expressed as the number of hours players slept the night before. Wellness and sleep variables were reported every morning throughout the study through a smartphone application (PM Reporter Pro). This application is a part of the Players Monitoring System (PMSys), an online sports logging system where athletes can track subjective wellness, training load, sickness and injuries [33]. All players were previously familiarized with the questionnaires, for at least five months.

### Data analysis and statistical methods

Data preparation and analysis was performed using the Tidyverse package [34] as well as the Ordinal package [35] in R software, version 4.2.2 [36].

To appropriately model the ordinal nature of the wellness variables, which were measured on a Likert scale, a cumulative link mixed model (CLMM) via the ‘clmm’ function from the ‘ordinal’ package in R was utilized. Preliminary data exploration indicated that the wellness variables were not normally distributed. Consequently, a probit link function (fitted via Laplace approximation) was selected within the models to accurately estimate the thresholds that delineate the ordinal categories. Given the presence of repeated measurements in our dataset and to ensure the independence of observations required for regression analysis, random slopes for individual subjects were incorporated. This adjustment helps prevent the inflation of p-values that could arise from the correlated data. To assess the impact of match days on wellness, five separate CLMMs were constructed. Each model treated a different wellness variable as the dependent variable and included match day status (dummy coded to compare MD, MD+1, and MD+2) as the predictor. After excluding missing data, the number of observations in the models ranged from 398–404 observations. Statistical significance was evaluated by reporting the estimates, standard error (SE), z-values, and p-values for each coefficient in the models. The conditional (fixed and random effects) and marginal (fixed effects) R^2^ values were also calculated.

The same rationale regarding ordinal variables was used for the correlation analysis between match load and wellness variables, which were computed using Spearman rank correlations to account for ordinal data. Correlations were interpreted as small (0.30–0.50), medium (0.50–0.7) and strong (>0.70). Additionally, the correlation analysis between match load variables and MD+1/MD+2 was computed separately.

## Results

**Fig 1:**
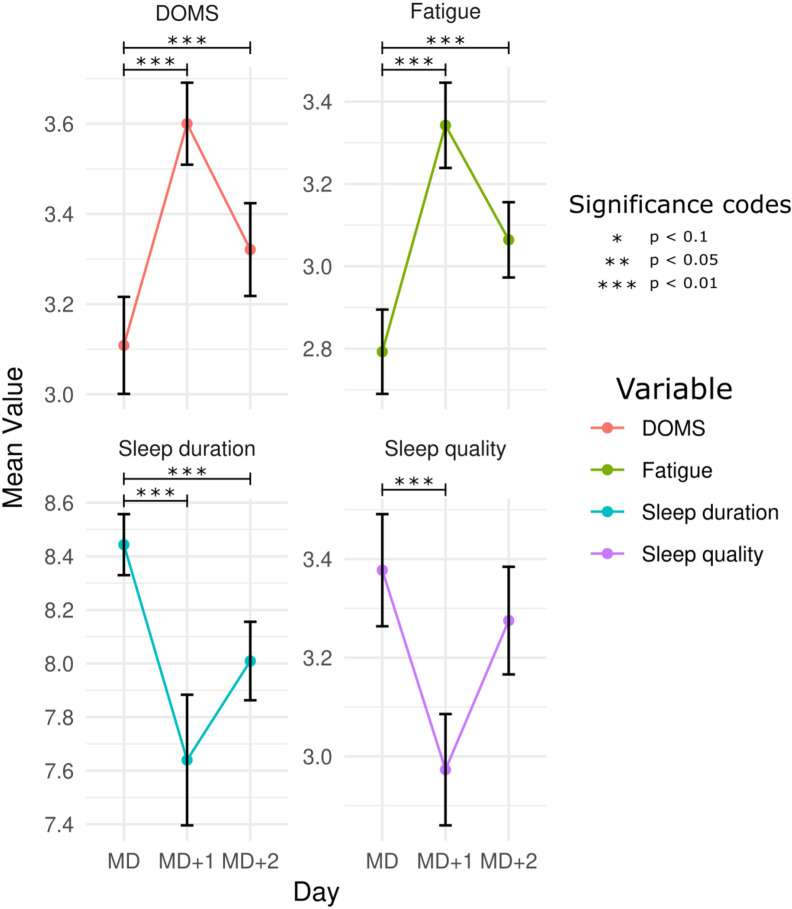
Average wellness for matchday, MD +1 and MD+2.

General descriptive statistics of match external load, players self-reported wellness and sleep duration are presented in Table 1. Significant differences in fatigue, sleep quality, sleep duration and DOMS between MD, MD+1 and MD+2 were observed and are illustrated in Fig 1. An increase in fatigue and DOMS was observed on MD+1 and MD+2. While sleep quality decreased on MD+1, sleep duration decreased on MD+1 and MD+2, when compared to MD.

**Table 1:** Descriptive statistics (mean ± standard deviation; minimum, median and maximum value) of included variables.

	Mean	Min	Median	Max
TotDist	9691.2 (±846.7)	8011.5	9611.0	11545.6
HSRD	1514.4 (±519.4)	510.5	1526.1	2974.7
SpD	377.9 (±196.3)	32.5	352.9	866.8
Acc_dist_	861.2 (±225.8)	432.1	838.3	1492.8
Sprints	32.3 (±14.7)	4.0	32.0	71.0
Fatigue	3.1 (±0.7)	1.0	3.0	5.0
DOMS	3.3 (±0.7)	1.0	3.0	5.0
Stress	2.9 (±0.5)	1.0	3.0	4.0
Sleep quality	3.1 (±0.7)	1.0	3.0	5.0
Sleep duration	8.1 (±1.2)	1.0	8.0	11.0

TotDist (Total Distance), HSRD (High-speed running distance), SpD (sprint distance) and Acc_dist_ (Acceleration distance) presented in meters. DOMS (Delayed Onset Muscular Soreness), Stress, Fatigue. And Sleep quality presented in arbitrary units. Sleep duration presented in hours.

The conditional R^2^ values varied from 0.251 in sleep duration to 0.527 in DOMS, indicating that the models explain between 25.1% and 52.7% of the variance. Conversely, the marginal R^2^ values ranged from 0.001 in stress to 0.168 in fatigue, suggesting that fixed effects contribute variably across models and that a substantial portion of the variance is attributable to individual differences. These findings underscore the significant role of individual differences in explaining the variability in wellness subsets, effectively captured by including random intercepts.

The results reveal significant increases in DOMS severity on MD+1 with an estimate of 1.11 (SE=0.16; z=7.1; p<0.001) and on MD+2 with an estimate of 0.49 (SE=0.15; z=3.3; p<0.001). These findings indicate that DOMS severity peaks on MD+1 and remains elevated but to a lesser extent on MD+2. It was also observed an increase in fatigue on MD+1 (estimate=1.3; SE=0.16; z=8.3; p<0.001) and MD+2 (estimate=0.75; SE=0.15; z=5.0; p<0.001). As with the DOMS, these results suggest that fatigue is most pronounced on MD+1 and remains elevated on MD+2.

An opposite pattern was observed for sleep quality. Our analysis revealed a significant decrease in sleep quality on MD+1 with an estimate of 0.72 (SE=0.14; z=5.1; p<0.001). However, on MD+2, the effect was smaller and non-significant (estimate=0.21; SE=0.14; z=1.5; p=0.129). The results observed also indicate a significant reduction in sleep duration on MD+1 (estimate=-0.70; SE=0.13; z=-5.4; p<0.001) and on MD+2 (estimate=-0.61; SE=0.13; z=-4.7; p<0.001).

Regarding the stress levels, the coefficients slightly reduced, though non-significantly, on MD+1 (estimate=-0.10; SE=0.16; z=-0.6; p=0.547) and MD+2 (estimate=-0.02; SE=0.16; z=-0.13; p=0.897).

**Fig 2:**
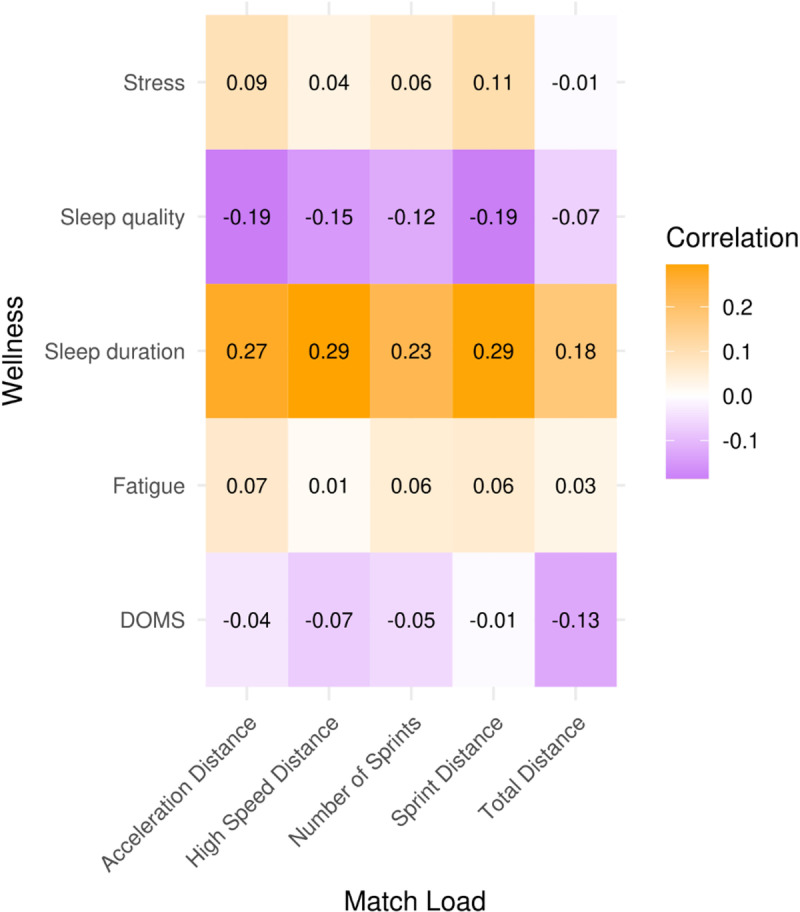
Correlation between match load and wellness on MD+1.

**Fig 3:**
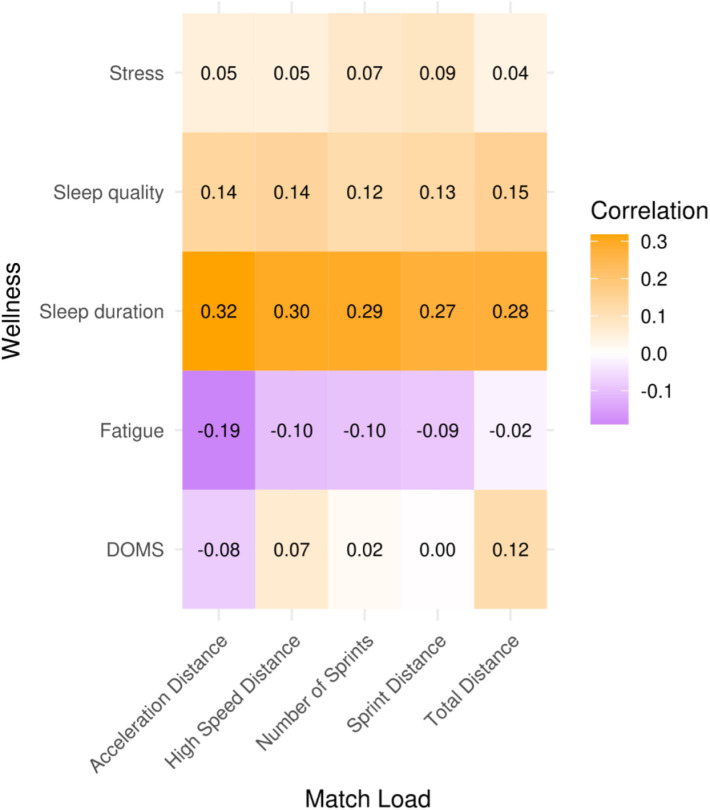
Correlation between match load and wellness on MD+2.

Correlation values between match external load metrics and MD+1 and MD+2 wellness variables are presented in Fig 2 and Fig 3. The highest correlation magnitudes (Spearman’s ρ) were observed on MD+2 between sleep duration and Acc_dist_ (0.32; moderate), HSRD (0.30; moderate), number of sprints (0.29; small), TotDist (0.28; small) and SpD (0.27; small). Other small and trivial correlations are reported, though with magnitude values below 0.25.

## Discussion

The current study investigated the influence of match physical demands on players’ perceived wellness on the two days following the match in highly trained female football players from two top-tier clubs. The main finding was that sleep duration and wellness subjective reports, such as fatigue, sleep quality and DOMS are significantly affected by competitive matches, with the stability of these measures being disrupted during the two days following the match. These results partly confirm the first hypothesis that wellness variables and sleep duration vary significantly from MD to MD+1 and MD+2. In fact, stress was the only variable analysed that was not sensitive to match demands, remaining stable between MD the following days.

These variations of the wellness scores in the days following competition were partly expected since previous research with men has revealed that high-intensity physical activity, such as a football match, requires up to four days to recover [37]. Moreover, a recent study with female football players [25] reported a disruption in the players’ sleep duration after an excessive training load, which is in line with the results of the present study, where sleep patterns (sleep quality and duration) were affected after a match. However, this variation seems to be lower on MD+2, where despite sleeping fewer hours (8.00 ±. 0.96 h) than on MD (8.50 ± 0.75 h), the quality of sleep (3.21 ± 0.71) returned to the pre-match values (3.31 ± 0.74). In this regard, even in studies where self-reported rankings were not sensitive to the variation of training load throughout the week (except for MD+1), the sleep quality category was the only exception [22]. This is an undesirable effect as good sleep indices are imperative for adequate recovery, and high match loads elevate the need for a proper recovery [38]. These disruptions in sleep patterns have previously been associated with a lack of sleep hygiene practices (e.g., late bedtime) [25], match-day routines (e.g., travelling and late treatments) [39], and physiological factors (e.g., elevated core temperature and high heart-rate) [40].

Similarly, fatigue and DOMS also presented a higher increase from MD to MD+1 compared to MD+2, reinforcing the idea that the most critical period for the players’ wellness is the 24 hours following a match. This variation in the levels of fatigue after competition was also observed in previous research with rugby players [41], revealing a temporary decrement in the players’ physical performance, which is likely derived from a reduction in neuromuscular function [42] and increased muscle damage [43]. Thus, albeit still significantly different, the wellness scores start to return to normal (pre-match values) on MD+2. The non-significant variation of the stress values from MD to MD+1 and MD+2 is corroborated by Fernandes et al. [44] where the authors concluded that football matches do not sufficiently trigger stress factors. Nevertheless, these results reinforce the importance of providing adequate recovery time post-match to minimize fatigue accumulation during the season and the consequent related pitfalls (e.g., illness, injury) [45]. Thus, coaching staff are advised to be cautious and induce very low training loads on the two days following a match. This strategy seems to be already common in men’s and women’s football [46], particularly for starters [6]. However, other recovery interventions and habits, such as individualized sleep hygiene and specific nutritional strategies (e.g., consuming some forms of protein prior to sleep), have been proposed for professional football players [47].

A previous multi-sport systematic review [16] revealed a small-to-moderate correlation between wellness measures and locomotor activity demands. In this way, our second hypothesis was that high-speed metrics accumulated during MD were correlated with the self-reported wellness and sleep duration of MD+ 1 and MD+ 2. However, the results of the present study do not corroborate the previously mentioned hypothesis. None of the wellness variables analysed were revealed to be highly correlated with the match external load. Perhaps stronger correlations could be observed if the match external load was analysed as a multi-variable construct. Only small and trivial correlations were observed on MD+ 1 between sleep quality and SpD (0.19), DOMS and TotDist (0.13), fatigue and Accdist (0.07), and stress and SpD (0.11). These results are in line with other research in women’s football [24], where post-match fatigue and DOMS ratings were not affected by substantial within-player variations in high-speed distances.

A few positive and moderate correlations were identified between wellness subsets and external load variables, and all of them were associated with sleep duration. The results reveal that despite being significantly affected by the match (disruption in sleep patterns), sleep quality and duration are somewhat correlated with specific load metrics. Altogether, this may indicate a tipping point caused by extremely high match loads where the tiredness and exhaustion experienced by the players lead to a lower disruption of sleep patterns. However, further research on sleep patterns is needed to corroborate and better explain these correlations. These results partly diverge from previous research, where no significant correlations were found in male players between training load and subjective sleep [48] and where inverse relationships between training loads and sleep duration were reported for female players [25]. A recent systematic review [49] has mentioned these inconsistencies in the literature on the relationship between training/match loads and sleep patterns. However, the results of the present study should be considered as the desired outcome due to the critical importance of sleep in the recovery process [50], as high loads imposed by training sessions and matches increase the need for recovery and, consequently, a better sleep hygiene [38,39]. The sleep variations observed in the present study may also reveal a lower sleep efficiency of the players involved [51]. Altogether, and in line with the conclusions of Scott et al. [24], the results revealed a lack of dose-response relationship between subjective wellness and external match load, which raises questions regarding the utility of simultaneously applying these measures in practice.

Moreover, our analysis shows that it is crucial to account for individual differences in responding when conducting this type of study, due to how data is collected. There is minimal variance to the model, with almost everyone responding 3, and it is not possible to respond 2.5 or 3.5, for instance. This was especially true when it comes to stress, where only 1% of the variance in stress was explained by which day it was, and the remaining 40% was due to individual differences.

To our knowledge, only two studies have investigated positional differences in wellness measures with male football players [52,53] and only one with female football players [54]. While one study reported differences in sleep quality between goalkeepers and defenders [52], no differences across playing positions were observed in the other two studies. Despite not being a specific aim of the present study, and due to its recognised importance for practitioners, the variations of wellness measures in outfield positions were briefly analysed. The preliminary results suggest that Central Forward is the playing position that mainly differs from the others, presenting different patterns of DOMS, stress, sleep duration and sleep quality from MD to MD+1 and MD+2. Besides that, the results also reveal that Fullbacks present very similar patterns among all the wellness variables analysed. However, despite the preliminary results described here, further studies and larger samples are needed to draw stronger conclusions since the sample used in the present study revealed an unequal distribution of the players involved across the different playing positions.

### Limitations and further research

In the present study, the sample size (18 players/ 175 match observations) impairs a generalization of the results since actual differences can be masked due to a statistical type 2 error, and such a consequence cannot be fully ruled out. This is a common limitation within the literature when studying professional players, particularly when involving female athletes. Even so, to increase data heterogeneity and diminish the risk of bias, the suggestion of previous research [55] for the importance of conducting multi-club studies by including two different top-level clubs was followed. Nevertheless, future studies should attempt to establish multi-club or multi-national collaborations where larger cohort studies may be conducted. This strategy is of utmost importance to assure the results’ generalizability.

Some lifestyle factors and contextual variables, such as match results, travel schedules and stage of the menstrual cycle, were not considered for the analyses and may warrant further investigation. This information may be necessary for interpreting the results since longer travel distances associated with negative results may contribute to worse sleep quality and increased fatigue [56], as well as the stage of the menstrual cycle, which may affect the readiness to train and perform [57]. Such analysis would be relevant for future studies to analyse if the players’ match performance and wellness scores on MD+1 and MD+2 are affected by the hormonal fluctuations during the different stages of the menstrual cycle, consequently providing valuable information regarding the risk of injury during periods of greater susceptibility. These unmeasured variables may partly explain the lack of strong correlations in the present study. However, since such data was not collected in our study, future research that considers these factors is warranted.

Another eventual limitation could be that the players’ wellness status was obtained by a simple 1–5 Likert scale questionnaire while more detailed and longer multi-question tools are available in the literature [23]. Moreover, despite being commonly used, the validity and reliability of this self-reported approach have not been rigorously evaluated [58]. Notwithstanding, these longer inventories may take up to 4 min of completion time, which is impractical to implement daily in any level of practice [59]. Conversely, wellness scales with shorter completion times (<1 min), such as the one used in the present study, are easier to implement and likely warrant a higher degree of adherence from the athletes. Additionally, as it fell out of the scope of the present study, training load was not collected, and further studies should explore the long-term effects of cumulative loads over a season. Apart from that, research on gender-specific recovery strategies is also required as the current findings may not generalize to male athletes.

Lastly, in the questionnaire suggested by McLean et al. [32], another subjective variable (i.e., Mood) is included. However, several constraints are inherent to using real football teams in research, and therefore, its applicability was considered non-feasible for the present study.

Altogether, the results of the present study highlight the importance of adequately monitoring training load and players’ wellness. With this information, practitioners may proactively intervene during specific stages of training and competition by individually adjusting recovery strategies and prescribing training loads according to the progress of the players’ recovery state while simultaneously attempting to maximize or maintain players’ performance. Ideally, the outcomes of any research should be a tool to inform practitioners in their decision-making. However, readers may consider their needs when deciding which athlete self-reporting measures to evaluate and prioritize. Finally, it is also important to remember that self-reported measures are predominantly used as status indicators and communication channels while their practical utility for training purposes needs further investigation [16].

## Conclusion

The present study sheds light on the variation of self-reported wellness variables from MD to MD+1 and MD+2. It was observed that competitive matches are associated with disrupting the stability of the players’ sleep patterns and wellness. Additionally, any of the wellness variables analysed were revealed to be highly correlated with a specific metric of match external load. These results suggest that univariate measures may not be strong enough to predict the players’ wellness status variation in the following days. Nevertheless, higher correlations were observed on MD+2 than on MD+1, though only positive and moderate correlations were identified, and all associated with sleep duration.
